# DNA reference reagents isolate biases in microbiome profiling: a global multi-lab study

**DOI:** 10.1128/msystems.00466-25

**Published:** 2025-10-17

**Authors:** Saba Anwar, Matthew Lamaudiere, Jack Hassall, Jacob Dehinsilu, Ravneet K. Bhuller, Georgina L. Hold, Xabier Vázquez-Campos, Alexander Mahnert, Christine Moissl-Eichinger, Birgit Gallé, Gudrun Kainz, Petra Pjevac, Bela Hausmann, Jasmin Schwarz, Gudrun Kohl, David Berry, Sarah J. Vancuren, Emma Allen-Vercoe, Nynne Nielsen, Nikolaj Sørensen, Aron Eklund, Henrik Bjørn Nielsen, René Riedel, Jannike Lea Krause, Hyun-Dong Chang, Suenie Park, Ho-Yeon Song, Hoonhee Seo, Asad ul-Haq, Sukyung Kim, Yongbin Kwon, Sunwha Park, Xavier Soberon, Eugenia Silva-Herzog, Joost A. M. Verlouw, Pascal Arp, Mila Jhamai, Robert Kraaij, Anoecim R. Geelen, Quinten R. Ducarmon, Wiep Klaas Smits, Ed J. Kuijper, Romy D. Zwittink, Niels van Best, John Penders, Giang Le, Christel Driessen, Jolanda Kool, Sudarshan A. Shetty, Susana Fuentes, Mehmet Demirci, Akin Yigin, Celina Whalley, Andrew D. Beggs, Christopher Quince, Rob James, Sebastien Raguideau, Martin Gordon, Ryan Mate, Martin Fritzsche, Nathan P. Danckert, Jesus Miguens Blanco, Julian R. Marchesi, Marcus Rauch, R. Anthony Williamson, Angélique B. van't Wout, Angelika Kritz, Stephan Rosecker, Richard Stevens, Lynette Laws, Lizbeth Sayavedra, Stefano Romano, Andrea Telatin, David Baker, Arjan Narbad, Stephanie L. Servetas, Jason G. Kralj, Samuel P. Forry, Monique E. Hunter, Jennifer N. Dootz, Scott A. Jackson, Christopher E. Mason, Daniel J. Butler, Christopher Mozsary, Jonathan Foox, Namita Damle, Aidan Resh, Amanda Busswitz, Peter Lenz, Shane Sontag, Andrew Cross, Christian Sanchez, Mingsheng Guo, Kayla Olson, Eric A. Smith, Alex J. La Reau, Tonya Ward, Scott Kuersten, Fred Hyde, Irina Khrebtukova, Gary Schroth, Sjoerd Rijpkema, Gregory C. A. Amos, Chrysi Sergaki

**Affiliations:** 1 Medicines and Healthcare Products Regulatory Agency 9059 , London, United Kingdom; 2 UNSW Microbiome Research Centre, St George Hospital 4335, Kogarah, New South Wales, Australia; 3 NSW Systems Biology Initiative, School of Biotechnology and Biomolecular Sciences, University of New South Wales 98492, Sydney, New South Wales, Australia; 4 Diagnostic & Research Institute of Hygiene, Microbiology and Environmental Medicine, Medical University of Graz 670030, Graz, Austria; 5 BioTechMed-Graz 600944 , Graz, Austria; 6 Core Facility Molecular Biology, Center for medical research, Medical University of Graz 569183, Graz, Austria; 7 Joint Microbiome Facility of the Medical University of Vienna, University of Vienna 27258, Vienna, Austria; 8 Department of Microbiology and Ecosystem Science, Centre for Microbiology and Environmental Systems Science, UBB University of Vienna 418299 , Vienna, Austria; 9 Department of Laboratory Medicine, Medical University of Vienna 27271, Vienna, Austria; 10 Department of Molecular and Cellular Biology, University of Guelph 317113, Guelph, Ontario, Canada; 11 Clinical Microbiomics A/S, Copenhagen, Denmark; 12 German Rheumatology Research Center Berlin, A Leibniz Institute, Berlin, Germany; 13 Bioinformatics and Computational Biology, University Heart & Vascular Centre Hamburg, University Medical Centre Hamburg-Eppendorf 37734, Hamburg, Germany; 14 Chair of Cytometry, Institute of Biotechnology, Technische Universität Berlin 672110 , Berlin, Germany; 15 Probiotics Microbiome Convergence Center, Soonchunhyang University 37969, Asan-si, South Korea; 16 BiowaveW, Seoul, South Korea; 17 Department of Obstetrics and Gynecology, Ewha College of Medicine 92203, Seoul, South Korea; 18 Unidad de Vinculación Cientifica UNAM-INMEGEN, Instituto Nacional de Medicina Genomica 7180, Mexico, Mexico; 19 Instituto de Biotecnologia UNAM 42560 , Cuernavaca, Morelos, Mexico; 20 Laboratory of Population Genomics, Department of Internal Medicine, Erasmus University Medical Center 273225 , Rotterdam, the Netherlands; 21 Center for Microbiome Analyses and Therapeutics, Department of Medical Microbiology, Leiden University Medical Center 541118, Leiden, the Netherlands; 22 Department of Medical Microbiology, Infectious Diseases and Infection Prevention, School for Nutrition and Translational Research in Metabolism, Maastricht University 385783, Maastricht, the Netherlands; 23 Center for infectious disease control, Dutch National Institute for Public Health and the Environment 10206, Bilthoven, the Netherlands; 24 Department of Medical Microbiology, Faculty of Medicine, Kırklareli University 187469, Kırklareli, Turkey; 25 Department of Genetics, Faculty of Veterinary Medicine, Harran University 52966, Şanlıurfa, Turkey; 26 Genomics Birmingham, Institute of Cancer & Genomic Sciences, University of Birmingham 152870, Birmingham, United Kingdom; 27 Earlham Institute and Quadram Institute Bioscience 150238, Norwich, United Kingdom; 28 Imperial College London 4615, London, United Kingdom; 29 AlphaBiomics Ltd., London, United Kingdom; 30 Source BioScience UK LTD 128692 , Nottingham, United Kingdom; 31 Quadram Institute Bioscience-Gut Microbes and Health Research Programme 7308, Norwich, United Kingdom; 32 National Institute of Standards and Technology 10833, Gaithersburg, Maryland, USA; 33 Department of Physiology and Biophysics, Weill Cornell Medicine 344068 , New York, New York, USA; 34 Diversigen, Inc., New Brighton, Minnesota, USA; 35 Illumina, Inc. 10908, San Diego, California, USA; Cleveland Clinic, Cleveland, Ohio, USA

**Keywords:** gut microbiome

## Abstract

When profiling the human gut microbiome, technical biases introduced by analytical approaches impede translational research, reducing data reliability and study comparability. Here, through a global study involving 23 labs, we analyzed a wide range of sequencing and bioinformatic approaches for the taxonomic profiling of two well-defined DNA reference reagents (RRs) comprised of 20 common gut bacteria. Through both shotgun and 16S rRNA gene amplicon sequencing, we aimed to isolate sources of bias and understand their impact on microbiome profiling accuracy. Importantly, minimum quality criteria (MQC) were established and are used to evaluate profiling performance. We found that the variability of shotgun sequencing data sets was greater than that of 16S rRNA gene amplicon sequencing and isolated sources of bias in wet and dry lab steps, such as sequencing depth, primer and database choices, rarefaction, and 16S copy number adjustment. This study presents well-defined RRs and MQC to combat technical bias, paving the way for reliable and comparable microbiome research.

**IMPORTANCE:**

This benchmark paper highlights the true level of variability in microbiome data across the world and across sectors, underscoring the critical need for the use of WHO International DNA Gut Reference Reagents (RRs) to elevate the quality of data in microbiome research. This global study is the first of its kind, revealing the reality of the bias in the field, comprehensively testing methodologies used by leading laboratories across the world, but also providing avenues for workflow optimization, to accelerate innovation and translational research and move the field forward.

## INTRODUCTION

Developments in next-generation sequencing (NGS) technologies have supported the rapid expansion of the microbiome field. However, there is currently no consensus surrounding methodology, and the variety of methodologies has led to inconsistent data and uncertainty on best practices. Contradictory findings are common when using different methods to analyze similar or identical samples (1–4). Microbiome studies inherently face bias, such as during sample collection, storage, DNA extraction, library preparation, and sequencing. This leads to inaccurate reporting of microbiome sample composition. To address bias and improve results, literature reports and thought leaders call for standardization of NGS protocols to aid translational research and product development (3, 5–7).

For reliable comparisons and reproducibility across microbiome studies, a well-characterized reference reagent to act as a global reference point serving as ground truth is crucial. Developing this material for sequencing and data analysis (downstream) will ensure high-quality data and allow for accurate identification of biases in earlier steps (upstream) of the workflow. While commercial microbiome standards exist, and reference biological samples (e.g., fecal slurry) could be used, we have previously shown that different reference reagents (RRs) have limitations (4). Community efforts have been made to address these biases using a similar approach, revealing variability at every step of the process (1). The study presented here is distinct in that it goes one step further, providing World Health Organization (WHO) International Reference Reagents validated by the community that can be employed to further expand on the findings of this study, standardize current methodologies and ensure commutability of data across the world. By employing a well-defined ground truth, combined with a stepwise methodology encompassing sequencing and bioinformatics analysis and isolating the process from external factors, this strategy provides a systematic framework for evaluating variation at each stage. It is therefore imperative to have purpose-specific reagents, which attempt to simulate the complexity of the target sample, to reveal bias, ensuring that the methods used provide meaningful results. Ultimately, global harmonization of data cannot be achieved so long as different studies use different standards, so the WHO International Reference Reagents serve as that global reference point ensuring reproducibility and comparability across studies, as well as a primary calibrant.

The WHO International Reference Reagents are primary standards, sometimes referred to as “gold” standards, and their use as a single primary reference reagent worldwide facilitates comparability of results across studies (8). The need for reference reagents with an official WHO status is often identified as a result of both regulatory and scientific activities.

The Microbiome group at the Medicines and Healthcare products Regulatory Agency (MHRA) developed the DNA-Gut-Mix (NIBSC 20/302) and the DNA-Gut-HiLo (NIBSC 20/304) reference reagents for gut microbiome analysis by NGS (4). These materials mimic the human gut microbiome and allow researchers to challenge the sensitivity and specificity (the ability to attribute individual reads to the correct organism) of pipelines. A WHO international collaborative study was conducted to evaluate these reference materials using various NGS techniques, ensuring representation from academic institutions, governmental bodies, service providers, and the pharmaceutical industry. The reporting system as described in reference 4, was used to set minimum quality criteria (MQC) to guide the quality assessment of metagenomic data. These RRs have been evaluated by the WHO Expert Committee for Biological Standardization (ECBS) and have been established as the first WHO International Reference Reagents for Gut Microbiome analysis by NGS (9). The reagents are sold not for profit, on a cost recovery basis, ensuring accessibility across the world (https://nibsc.org/products/brm_product_catalogue/sub_category_listing.aspx?category=Diagnostics&subcategory=Microbiome).

The aim of this study was to identify the utility of the DNA-Gut RRs in revealing variability and bias introduced by sequencing and bioinformatics during microbiome taxonomic profiling, understand the real level of variability in analysis across the world, and to establish MQC for users based on real-life data. This study can therefore serve as a benchmark for assessing the evolving analytical performance of future microbiome profiling tools over time. In doing so, we aim to raise the quality, reproducibility, and trust in microbiome data and techniques across the world, ultimately leading to a better understanding of the human gut microbiome.

## RESULTS

### Variability in shotgun sequencing analyses across the world

**Fig 1:**
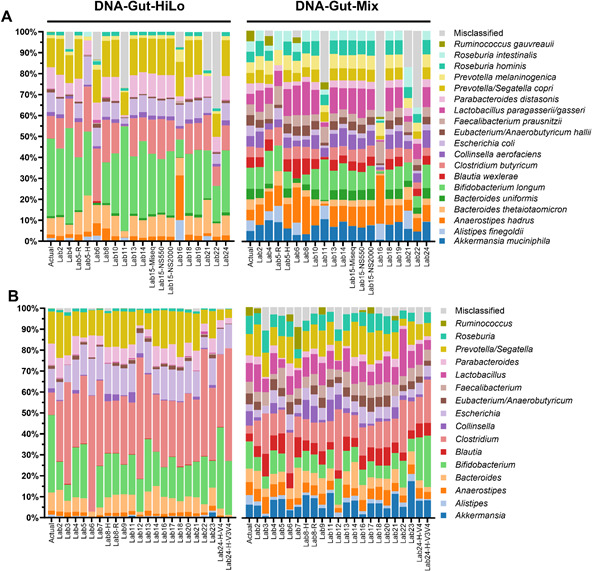
Average microbiome composition for each method employed by the participants (*n* = 5), aligned to the ground truth (Actual) of each DNA RR. (**A**) Relative species level composition (%) of the DNA-Gut-HiLo and DNA-Gut-Mix RRs resulting from shotgun sequencing data analysis. (**B**) Relative genus level composition (%) of the DNA-Gut-HiLo and DNA-Gut-Mix RRs resulting from 16S rRNA gene amplicon sequencing data analysis.

To assess the variation in shotgun sequencing data (Fig. 1) the four key reporting measures were assessed for both RRs. Across the 19 shotgun sequencing data sets for the DNA-Gut-Mix RR, sensitivity ranged from 74% to 100%, false positive relative abundance (FPRA) ranged from 0% to 41%, diversity ranged from 14 to 180 species, and similarity ranged from 34% to 87% (Table 1). For the DNA-Gut-HiLo RR, sensitivity ranged from 63% to 100%, FPRA ranged from 0% to 13%, diversity ranged from 12 to 185 species, and similarity ranged from 55% to 95% (Table 1). The DNA-Gut-HiLo RR served to challenge the analytical sensitivity of participant methodologies more so than the DNA-Gut-Mix (*P* value = 0.0208), which in many cases was unable to identify the low-abundant species.

**TABLE 1:** The four key reporting measures for the different shotgun sequencing data sets, as calculated using species-level taxonomic data^*a*^

Data set	Sensitivity (%)		FPRA (%)		Diversity		Similarity (%)	
	HiLo	Mix	HiLo	Mix	HiLo	Mix	HiLo	Mix
Actual	**100**	**100**	**0**	**0**	**19**	**19**	**100**	**100**
Lab 2	**68**	89	**0**	**0**	**13**	17	**90**	**84**
Lab 4	**95**	**95**	8.06	2.57	21	23	**80**	**82**
Lab 5-H	**74**	74	**0**	**0**	**14**	14	71	68
Lab 5-R	63	89	**0**	**0**	12	17	**83**	**80**
Lab 6	**95**	**95**	13.36	3.25	**19**	**19**	60	71
Lab 8	**95**	**95**	**0**	**0**	**18**	**18**	**78**	**72**
Lab 10	**74**	**95**	**0**	**0**	**14**	**18**	**87**	**84**
Lab 11	**100**	**100**	1.06	2.84	170	180	69	68
Lab 13	**68**	**95**	**0**	**0**	**13**	**18**	**85**	**83**
Lab 14	63	**95**	**0**	**0**	12	**18**	**95**	**86**
Lab 15-MiSeq	63	**95**	**0**	**0**	12	**18**	**88**	**84**
Lab 15-NS2000	**68**	**95**	**0**	**0**	**13**	**18**	**89**	**81**
Lab 15-NS550	63	**95**	**0**	**0**	12	**18**	**83**	**85**
Lab 16	**95**	**95**	7.29	40.82	185	154	55	34
Lab 18	**95**	**95**	**0**	**0**	**19**	**19**	**88**	**85**
Lab 19	**95**	**95**	**0**	**0**	**19**	21	**91**	**86**
Lab 21	**84**	84	2.11	4.46	71	112	72	71
Lab 22	**95**	**95**	**0**	**0**	**18**	**19**	**77**	**77**
Lab 24	**95**	**95**	**0**	**0**	**19**	**19**	**90**	**87**
**MQC**	**≥68**	**≥95**	**≤0.53**	**≤1.29**	**13–19**	**18–19**	**≥75**	**≥72**

^*a*^ Including the minimum quality criteria (MQC). Measurements in bold meet the MQC. R, recommended, and H, in-house.

It is important to identify any common methodological features (Table S2) that influenced the observed key reporting measures. Seven of the 19 data sets met the MQC for all reporting measures for both RRs (Labs 8, 10, 13, 15-NS2000, 18, 22, and 24; Table S2). Six data sets used 150–151 bp read length, while one used 300 bp, five of them used Nextera adapters (four Nextera Flex and one Nextera XT), while one used NEBNext Multiplex Oligos for Illumina (Index primer sets one and two). Labs 13 and 15 used the recommended bioinformatics pipeline, while Labs 8 and 24 employed similar approaches to the recommended. Notably, Lab 10 reached the MQC with approximately 890 thousand reads per sample, substantially lower than the other five labs (averaging 25.4 million reads per sample), indicating that high-accuracy taxonomic profiling can be achieved with lower read depth.

**Fig 2:**
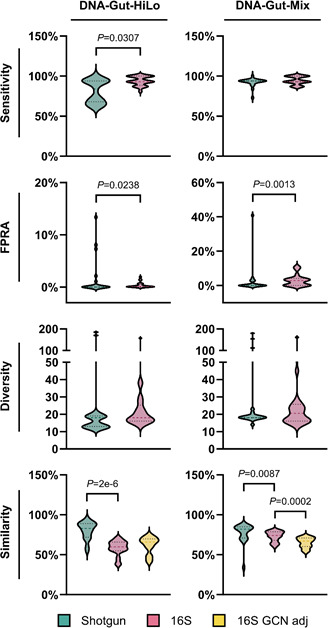
Comparison of key reporting measures from shotgun, 16S rRNA gene (16S), and 16S rRNA gene copy number adjusted (16S GCN adj) amplicon sequencing data sets for both DNA-Gut-HiLo and DNA-Gut-Mix reference reagents. Statistical analysis by Mann-Whitney *U* tests.

While shotgun sequencing offers a broader coverage and higher resolution of microbiome composition, 16S rRNA gene amplicon sequencing remains a cost-effective profiling tool for low-biomass samples or those with high levels of contaminant DNA. In this study, while shotgun sequencing allows for species-level analysis, data sets were found to have a greater coefficient of variation when compared to that of 16S rRNA gene amplicon sequencing data (Fig. 1 and 2; Table S3).

### Impact of rarefaction on pipeline performance

We further analyzed these data to assess the impact of rarefaction of shotgun sequencing data on accuracy as this is often a topic of debate across the field (10–12). When the data were rarefied (subsampled to a depth corresponding to 0.005% of the total reads), similarity measures were not affected. Rarefaction mostly impacted diversity measures with an average reduction of 46% and 40% for DNA-Gut-Mix and DNA-Gut-HiLo RR, respectively, removing up to 142 species in one case (Table S4). Despite improving specificity by reducing false positives, rarefaction decreased sensitivity measures for three data sets, highlighting the sensitivity-specificity trade-off as previously shown (13). Through these RRs, users can decide if rarefaction is suitable for their data set, proving valuable for approaches that greatly overestimate sample diversity.

### Impact of database choice on pipeline performance

Database choice significantly affected results. All three Lab 15 data sets did not identify *Alistipes* in either RR (using ChocoPhlAn3), while Labs 11 and 16 detected additional *Collinsella* spp. (8 and 4 additional species, respectively) not present in the RRs (Table S5), likely misclassifying reads from *Collinsella aerofaciens* sequences. No data set produced using the MetaPhlAn3 database (ChocoPhlAn3) identified *Ruminococcus gauvreauii*, as the species was absent from the database. The only shared analytical aspect between the two data sets to report the greatest overestimations of diversity, Labs 11 and 16 (Table 1), was database choice, namely, RefSeq (Venti) and RefSeq (Rep 82), respectively. It is noteworthy that continual reclassification of bacterial species can lead to outdated databases, disagreement between databases, and subsequent inaccurate taxonomic profiling, the same may also arise from the use of non-curated databases.

### Uncoupling sequencing and bioinformatics bias

A consistent library preparation and bioinformatics approach can be employed while allowing variation in sequencing approach to uncouple and pinpoint sources of bias in sequencing. Lab 15 sequenced the RRs using three different sequencing platforms and depths (Table S1), and by keeping the bioinformatics analyses consistent, we were able to identify differences in diversity and similarity measures (Table 1; Table S6). Deeper sequencing using NextSeq 2000 improved the similarity measure and allowed for the identification of *Lactobacillus paragasseri* (now distinct from *Lactobacillus gasseri*) in the DNA-Gut-HiLo RR, a species of low abundance, which was not found at lower sequencing depth. However, increasing sequencing depth alone cannot overcome database limitations. For instance, even at 286 million reads per sample, only 13 of 16 genera were identified in the DNA-Gut-HiLo RR. While higher read depth can help identify less abundant microbes, it does not always improve accuracy due to database constraints. Therefore, optimizing library preparation approaches is also crucial for enhancing accuracy. Hence, this approach can be expanded to optimizing library preparation approaches, which was also found to influence overall accuracy.

### Meta-analysis of shotgun sequencing data sets

**Fig 3:**
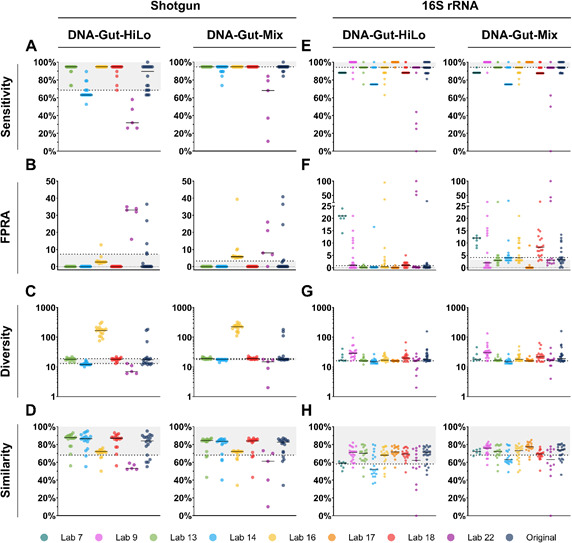
Participant meta-analyses of shotgun and 16S rRNA gene amplicon sequencing data sets performance recorded using the four key reporting measures. Comparison of (**A**) sensitivity, (**B**) FPRA, (**C**) diversity, and (**D**) similarity measures, recorded following meta-analyses of the DNA-Gut-HiLo and DNA-Gut-Mix RR shotgun sequencing data, compared to original participant analysis. Comparison of (**E**) sensitivity, (**F**) FPRA, (**G**) diversity, and (**H**) similarity measures, recorded following meta-analyses of DNA-Gut-HiLo and DNA-Gut-Mix RR 16S rRNA gene amplicon sequencing data, compared to original participant analysis. If data reside within the gray area of the graph, or the dotted line for 16S rRNA gene diversity, they meet the MQC for the given key reporting measure.

Meta-analysis of all data sets using consistent bioinformatics approaches (Table 2) can determine whether sequencing or bioinformatics (or a combination of the two) can influence overall accuracy, isolating bias introduced during different stages of the process. Meta-analyses by Labs 13 and 18 outperformed the initial analysis conducted by participants, resulting in higher overall accuracy (Fig. 3A through D), with Lab 13 doubling the number of labs originally meeting all MQC. Choice of bioinformatic pipeline can also decrease data set accuracy, reducing the number of labs meeting the MQC to zero in certain cases. During the meta-analysis, a trade-off between sensitivity and specificity was observed, as previously discussed. However, conservative approaches, such as the one used by Lab 14, can reduce both sensitivity and diversity, highlighting the fine balance needed to avoid the inverse. Notably, no data set analyzed by Lab 22 met the MQC, perhaps due to their genome-centric metagenomics workflow, including generation of representative metagenome assembled genomes (MAGs) which is not an appropriate tool for the analysis of non-MAG-based data sets. This could be due to bias toward genomes with the best coverage, allowing for more complete genome assembly and accurate classification. This shows that bioinformatics pipelines can greatly affect overall performance despite variation in sequencing approaches, indicating that by using RRs, it is possible in many cases to improve data accuracy by adjusting only the bioinformatic tools.

**TABLE 2:** Participant bioinformatics methods used for meta-analysis of shotgun sequencing data sets

Participant	Trimming	Pipeline	Database	Subsampling/rarefaction
Lab 13	BBDuk (v.38.90)	MetaPhlAn (v.3.0.9)	ChocoPhlAn3	Seqtk: 1.3, 250k fwd, 250k rev
Lab 14	BBDuk (v.38.90)	MetaPhlAn (v.3.0.9)	ChocoPhlAn3	Seqtk: 1.3, 250k fwd, 250k rev
Lab 16	Shi7 (default parameters)	BURST DNA aligner (default parameters)	RefSeq representative prokaryotic genome pre-built database collection (86)	No
Lab 18	BBDuk (v.38.90)	KneadData/HUMAnN3/MetaPhlAn3 (v.3.0.7)	ChocoPhlAn3	No
Lab 22	Atlas 2.8 snakemake	Atlas 2.8 snakemake	checkM, Dram, EggNOG (v5), GTDB (v6)	No

### Variability in 16S rRNA gene amplicon sequencing analyses across the world

Twenty-three 16S rRNA gene amplicon sequencing data sets were produced by participants (Table S7). For the DNA-Gut-HiLo RR data, sensitivity ranged from 81% to 100%, FPRA ranged from 0% to 1.91%, diversity ranged from 14 to 157, and similarity ranged from 38% to 71%. For the DNA-Gut-Mix RR, sensitivity ranged from 87% to 100%, FPRA ranged from 0% to 11.1%, diversity ranged from 14 to 161, and similarity ranged from 60% to 81% (Table 3). Intriguingly, when calculated using 16S gene copy number adjusted data, similarity measures increased by an average of 2.9% across all participants, for the DNA-Gut-HiLo RR, while decreasing by an average of 8% for the DNA-Gut-Mix RR (Table 3; Fig. 2). Despite this, the MQC does decrease when calculated with 16S rRNA gene amplicon sequencing data.

**TABLE 3:** The four key reporting measures for the different 16S rRNA gene amplicon sequencing data sets as calculated using genus-level taxonomic data^*a*^

Data set	Sensitivity (%)		FPRA (%)		Diversity		Similarity (%)		Similarity (16S—copy number adjusted) (%)	
	HiLo	Mix	HiLo	Mix	HiLo	Mix	HiLo	Mix	HiLo	Mix
Actual	100	100	**0**	**0**	**16**	**16**	**100**	**100**	**100**	**100**
Lab 2	**100**	**100**	0.65	**0.5**	18	18	**61**	**78**	**68**	**72**
Lab 3	81	88	1.91	11.08	17	24	43	60	49	53
Lab 4	**94**	**94**	**0.13**	**3.4**	21	20	**71**	**80**	**72**	**69**
Lab 5	**94**	**94**	**0.12**	4.1	**16**	**16**	**64**	**73**	**66**	**61**
Lab 6-P	**100**	**100**	0.64	**3.38**	37	53	38	**68**	42	**63**
Lab 6-S	**100**	**100**	0.66	**3.46**	39	56	38	**68**	43	**63**
Lab 7	**100**	**100**	**0.05**	9.42	26	45	**55**	64	**59**	55
Lab 8-H	88	88	1.29	6.39	17	15	**66**	**77**	**74**	**71**
Lab 8-R	**94**	**94**	**0.1**	**3.04**	**16**	**16**	**67**	**81**	**74**	**72**
Lab 9	**100**	**100**	**0.01**	**0.01**	23	22	**60**	**80**	**67**	**73**
Lab 11	**94**	**94**	**0**	**0**	19	21	**65**	**80**	**71**	**74**
Lab 12	88	88	**0.21**	10.51	18	23	47	62	41	57
Lab 13	**94**	**94**	**0.16**	**3.33**	**16**	**16**	**68**	**79**	**65**	**67**
Lab 14	**94**	**94**	**0.12**	**2.73**	**16**	**16**	**63**	**73**	**69**	**67**
Lab 16	**100**	**100**	**0.02**	**0.03**	21	26	**58**	**76**	**66**	**69**
Lab 17	**100**	**100**	**0.02**	**0.01**	30	25	**66**	**80**	**72**	**71**
Lab 18	**94**	**94**	**0.03**	**0**	22	17	**56**	**77**	**68**	**67**
Lab 20	**100**	**100**	**0.36**	**4.07**	28	28	**58**	**74**	**62**	**65**
Lab 21	**100**	**100**	1.48	4.14	157	161	**63**	**77**	**70**	**72**
Lab 22	**94**	**94**	**0.02**	**1.59**	**16**	**16**	**55**	64	48	59
Lab 23	**94**	**94**	**0.17**	**2.59**	**16**	**16**	**60**	**74**	**61**	**61**
Lab 24-V3-V4	88	**94**	**0**	**0.01**	15	17	**55**	64	48	55
Lab 24-V4	88	88	**0**	**0**	14	14	**71**	**71**	**59**	60
**MQC**	**≥94**	**≥94**	**≤0.50**	**≤4.09**	**16**	**16**	**≥55**	**≥68**	**≥54**	**≥61**

^*a*^ Including the minimum quality criteria. Measures in bold meet the MQC. R, recommended; H, in-house; P, paired-end, and S, single-end.

The four participants to achieve all MQC for both RRs (Table S8) used the MiSeq sequencing platform, paired-end reads, and a read length of 250–300 bp. Three participants chose the V4 region (515F/806R) targeting primers, one chose the V3–V4 (341F/806R) region, indicating that MQC can be met using both primer sets (Table S9). An array of sequencing depths (from 11,755 to 528,347 reads per sample) and bioinformatics pipelines were employed. On the other hand, of the data sets that do not meet all MQC for either RR, all but two overestimated sample diversity for at least one RR. Six data sets met the MQC for all measures, excluding diversity. This is due to misclassification of genera, contamination, either technical or artificial, and/or noise from, e.g., variation in instrumentation, sample preparation, and bioinformatics pipelines (14).

Lab 16 analyzed their sequencing data twice, using two versions of the SILVA database, v.138 and v.138.1. Only when using SILVA v.138.1 were *Eubacterium* and *Ruminococcus* genera correctly identified (Table S10). While the sensitivity increased with the latest version of the database, the diversity also increased (Table S11), highlighting again the sensitivity-specificity trade-off and demonstrating that database version alone can impact the accuracy of microbiome analysis. This marked database-specific discrepancy in performance using a community of only 16 genera highlights the magnitude of the potential influence of databases when studying large, complex microbial communities. However, the database choice alone does not guarantee better performance, as four of the five participants using SILVA v.138.1 did not meet all MQC for both RRs.

Lab 8 performed data analysis using two different bioinformatic pipelines, Dadaist2 and QIIME2 Deblur, while keeping the trimming and the database consistent. QIIME2 Deblur outperformed Dadaist2 across all key reporting measures. This is consistent with previous findings that pipeline choice alone can have a significant impact on microbiome analyses (4), an approach explored to a greater extent through participant meta-analyses.

### Meta-analysis of 16S rRNA gene amplicon sequencing data sets

All 16S rRNA gene amplicon sequencing data sets were also subject to meta-analysis, assessed with a consistent bioinformatics approach chosen by participants (Table 4; Fig. 3E through H). Labs 13 and 17 recorded improved key reporting measures compared to the original participant analyses, with Lab 17 doubling the number of data sets to meet all MQC for both RRs. The approach used improved diversity measures, a common pitfall of most other pipelines. Critically, Lab 22 used the same approach as Lab 13 with changes in certain quality parameters, resulting in a lower average performance across all reporting measures.

**TABLE 4:** Participant bioinformatics methods used for meta-analysis of 16S rRNA gene amplicon sequencing data sets

Participant	Trimming	Pipeline	Database	Subsampling/rarefaction
Lab 7	CutAdapt (v.3.4) and DADA2 (v.1.20.0)	DADA2 (v.1.20.0)/DECIPHER (v.2.20.0)	SILVA (v.138)	No
Lab 9	fastp (v.0.32.2) and DADA2	DADA2	SILVA (v.138.1)	No
Lab 13	CutAdapt/QIIME2 (NextFlow)	QIIME2 (v.2020.2) using Deblur (NextFlow)	SILVA (v.138)	No
Lab 14	CutAdapt/QIIME2	QIIME2 (v.2020.2) using Deblur	silva-138-99-nb-classifier.qza	No
Lab 16	DADA2 (v.1.18)	DADA2 (v.1.18)	SILVA (v.138)	No
Lab 17	CutAdapt (v.3.4)/DADA2 (v.1.20.0)/Biostrings	DADA2 (v.1.20.0)	SILVA (v.138.1)	No
Lab 18	DADA2 (v.1.18)	DADA2	SILVA (v.138.1)	No
Lab 22	CutAdapt/QIIME2	QIIME2 (v.2020.2)/DADA2 (modified^*a*^)	SILVA (v.138)	No

^*a*^ The modified version included the following; for denoising, forward reads were truncated at a length of 200 bp and reverse reads at a length of 150 bp. Twelve potential contaminants (based on processed negative controls) were identified in a per-batch mode with the prevalence method and a threshold of 0.5 with decontam (version 1.10.0) in R (version 4.0.3) (15) and removed by using the filter feature and sample functionalities of QIIME2.

Meta-analysis by Labs 7, 9, 16, 18, and 22 produced lower reporting measures than the original analyses. Labs 9, 16, 18, and 22 suffered from a sensitivity-specificity trade-off, with high sensitivity associated with overestimated diversity (and high FPRA). Labs 16 and 18 only differed in database versions, SILVA v.138 and v.138.1, respectively, yet Lab 18 reported a greater average diversity than Lab 16 by four additional genera for the DNA-Gut-HiLo and five for the DNA-Gut-Mix, confirming our earlier observations on the role of databases.

Rarefaction is the process of subsampling the reads of each sample to match the sample with the fewest reads, helping to normalize sequencing depth across different samples and enable fair comparisons of microbial taxonomic composition across samples. To understand the impact of rarefaction on the accuracy of the results, Lab 13 performed analysis with and without rarefaction to minimum sample depth for each data set. The overall impact of rarefaction was more pronounced for the DNA-Gut-HiLo than for the DNA-Gut-Mix RR; however, it appeared to be data set dependent and reduced the total number of data sets meeting all MQC for both RRs from 8 to 6 (Table S12), due to the reduction in similarity of the two affected data sets (Labs 22 and 24-V4) for the DNA-Gut-Mix RR. Interestingly, the similarity measures for the DNA-Gut-HiLo RR improved by an average of 4% (Fig. S1). This finding suggests that the decision-making process for the analytical approach should be informed through the use of RRs and be purpose specific.

## DISCUSSION

In this study, we aimed to understand the true level of variability in microbiome data produced by different methodologies across sectors and geographies, and to demonstrate how the RRs can help pinpoint sources of bias at different steps of the process. A set method was not prescribed to participants; rather, participants were free to perform a technique of their choosing, showcasing the real-world methods utilized by the field to characterize microbial composition. We intended to identify biases introduced during DNA sequencing and bioinformatics analysis of microbiome taxonomy. A wide range of techniques was employed to expose these biases and understand why they cause variation in reported data.

Using these diverse data sets, we established the MQC for each of the four key reporting measures to facilitate the assessment of data accuracy, elevating microbiome data quality worldwide. It is critical for the four key reporting measures to be used in combination and not in isolation, each of the four measures requires the context given by the others for a valid interpretation to be made. For example, meeting the MQC for sample diversity does not guarantee an accurate measure of diversity; false positives may be present, while what would be true positives may be missing. This would be evident by the presence of a non-null FPRA measure, which in isolation also gives limited insight into the accuracy of sample diversity. The Bray-Curtis dissimilarity measure is designed to allow for high-level inter-sample diversity comparisons across groups around the world and was chosen due to its simplicity and frequency of use in the field. It also does not rely on any phylogenetic components that add to the observed bias.

The MQC outlined in this study is representative of variable real-world data, rather than a theoretical threshold or prediction, and informed our conclusions throughout, allowing for direct comparisons and meta-analyses of data. This large study allowed us to test various long-standing controversies in the field, such as the importance of sensitivity-specificity trade-off, the value of rarefaction, the biases introduced by sequencing depth, primer and database choice, and adjusting for 16S gene copy number on overall profiling accuracy.

A greater degree of variation in participant performance was observed across all key reporting measures for both RRs in shotgun sequencing data compared to 16S rRNA gene amplicon sequencing data. The shotgun sequencing data may be more varied due to higher divergence between data processing approaches by each bioinformatics pipeline (16), coupled with the greater error rate associated with species level taxonomic assignment compared to genus level in 16S rRNA gene amplicon analyses (17). Taxonomic misclassification may stem from read error, algorithm error, database misidentification, or database omission (18, 19), ultimately reducing the accuracy of the reported taxonomy.

Consistent with previous findings, we identified a common trade-off between pipeline sensitivity and specificity, where increased sensitivity often led to high diversity and false positive reporting, for both shotgun and 16S rRNA gene amplicon analyses (20, 21). With the use of these RRs, users can understand the limitations of their approach and avoid over-prioritizing sensitivity at the cost of specificity. In both shotgun and 16S rRNA gene amplicon data sets, most false-positive species and genera were found to be closely associated with those present in the reagent, rather than environmental contaminants. Studies focusing on this observation could reveal whether this could be due to read errors, incorrect sequencing alignment, or other biases in the bioinformatics analysis, and whether certain species are more drawn to such errors with specific tools.

On the other hand, the value of rarefaction has long been debated in the field, with little consensus reached (10, 22). In this study, data rarefaction yielded inconsistent results when tested with an array of sequencing data sets and bioinformatic pipeline parameters. However, rarefaction had a positive effect on the overall accuracy of a few, primarily shotgun sequencing data sets, lowering diversity measures and reducing the impact of the sensitivity specificity trade-off. Hence, the data from this study argues that rarefaction can prove a useful tool, particularly if investigating rare/low-abundance microbes, but it may not always improve accuracy. The primary utility of rarefaction is to reduce type-1 errors and allow for more robust downstream statistical comparisons of samples (13). While not done for this study, it would be valuable to test how different rarefaction depths affect the accuracy of the data for different technologies, utilizing the RRs. Ultimately, the utility of rarefaction should be evaluated on a case-by-case basis using appropriate tools as outlined, following initial optimization.

Importantly, we identified database-specific biases. The impact of database choice on sample profiling was pronounced, with the lack of the *R. gauvreauii* genome in the ChocoPhlAn3 database affecting all key reporting measures for the shotgun sequencing approaches. This is striking as ChocoPhlAn3 is widely used and critically, *Ruminococcus* spp. can play a key pathological role in several human diseases, such as inflammatory bowel disease (23), metabolic syndrome (24), irritable bowel syndrome (25), and colorectal cancer (26). When analyzing this reagent following the conclusion of the study, with the updated version of the tool and database, MetaPhlAn4, this was corrected, while certain parameters seem to have been adjusted to also improve accuracy (9). This exemplifies that by pinpointing the sources of bias in existing methodologies using a reference reagent, we can push innovation and method optimization.

Furthermore, these 20-strain/16-genus RRs allowed us to observe the impact of 16S rRNA gene database version (SILVA v.138–v.138.1) on profiling performance. Changes in database versions alone had a positive effect by allowing the identification of previously omitted genera; however, it overestimated the sample diversity and increased false positive reporting. This is consistent with previous observations that database choice can impact the accuracy of taxonomic classification (19). Demonstrating this impact despite the limited complexity of the RRs highlights the extent of optimization needed in the field. Furthermore, controlling for database version and thinking critically about historical data is key to the progression of the field and bringing confidence to microbiome studies.

Meta-analysis of shotgun and 16S rRNA gene amplicon sequencing data sometimes improved key reporting measures by simply switching bioinformatic pipelines, highlighting the bioinformatic-specific biases. Yet, data sets that previously met all MQC (for at least one RR) were sometimes worsened to below the MQC with certain bioinformatics meta-analyses. Adopting different pipelines can improve profiling accuracy; however, it cannot overcome the limitations of lower quality or incompatible sequencing data. Ultimately, a bioinformatics pipeline can “make or break” a data set. Therefore, it is important to consider the biological basis of the biases introduced prior to analysis, such as the absence of certain bacterial species from the data set due to lower DNA extraction efficiency, as previously shown (27, 28). In such a case, bioinformatics pipelines cannot correct the biological bias, particularly when these biases are unknown or not yet fully understood. As such, the DNA-Gut-Hilo and DNA-Gut-Mix RRs should be used alongside the WHO whole-cell RR to identify biases at the earlier stages of the process, e.g., DNA extraction, to engender confidence in microbiome methodologies and data.

Besides the common biases identified in both shotgun and 16S rRNA gene sequencing approaches, we also revealed approach-specific biases. For shotgun sequencing, Lab 15 revealed that, as previously shown, sequencing platform choice, potentially due to different chemistry and/or sequencing depth capacity (29), can be a limiting factor for the overall accuracy of microbiome profiling. For 16S rRNA gene amplicon sequencing, the participants utilized eight different primer pairs for sequencing, allowing the comparison of data sets produced using different primer sets, often considered incomparable (30, 31). Here, we observed that V4-targeting primers more consistently produced accurate data; however, V3–V4 targeting primers met all MQC in one case. Therefore, primer selection alone does not account for all the observed bias. The two data sets produced using V1–V2 and V1–V9 primer pairs did not meet the MQC for either RR; however, these primers may be more suited to characterizing microbiomes of specific ecosystems, or even specific species in the human gut not present within the RRs (32, 33). Furthermore, while many argue the importance of adjusting abundance data to 16S rRNA gene copy number (GCN) (34, 35), when applied to this study, it worsened the pipeline performance. This could, in part, be a consequence of GCN prediction uncertainty, which has led to contradictory findings regarding the effectiveness of this method for metataxonomic analyses (36, 37).

This international blinded study using data from across the world has effectively revealed biases at various analytical steps and shed light on ongoing debates within the field. However, while highly informative, it has limitations. The employed reference materials do not fully recapitulate the complexities encountered with real-world samples, and as such, the accuracy of a given approach may decrease as sample complexity increases. Specifically, they do not account for the impact of, e.g., host contamination, sample heterogeneity, DNA from other resident microbes, or the presence of inhibitors that can all influence analytical pipeline performance. Additional standards assessing the effect of those parameters are still needed in the field to complement the existing standards and reveal their effect on data accuracy.

Furthermore, there is meaningful insight to be gained by investigating the specific pipeline parameters of each approach. This would require a systematic evaluation of each bioinformatic pipeline by altering single parameters between iterations to infer any given effect. Altering read filtering, trimming, denoising, normalization, rarefaction, mapping, and alignment parameters independently with the use of the RR as a baseline will help with pinpointing exact sources of bias. This could form the basis of a further, more granular study, and we encourage individuals to use the RRs in this way to optimize bioinformatic workflows. This study, however, is a valuable start, with the establishment of these RRs as a global reference point allowing us to track the accuracy of data sets across geographies, time, and generations of methods as the field progresses.

Importantly, this study can serve as a foundational baseline by which newly developed microbiome profiling tools can be evaluated, helping to benchmark their performance and reliability. This underscores the need for longitudinal studies and increases the long-term value of this study by encouraging validation and refinement of methodologies as the field evolves. Advocating for a standardized approach in the current landscape of microbiome profiling would only serve to hinder innovation, as currently, no single method or protocol can perform to an exacting standard, irrespective of sample composition. Ultimately, we advocate for a cautious approach to microbiome data interpretation, especially when comparing data sets. Labs across the world must consider the technical biases introduced by their methods and work to mitigate their impact with the appropriate use of RRs, alongside minimum reporting standards for microbiome studies, which should include comprehensive details of methodologies, pipeline parameters, and software versions employed. Inherent limitations of current methodologies must be acknowledged to ensure meaningful data interpretation and comparison.

### Conclusions

Here, we establish the WHO DNA-Gut-HiLo and WHO DNA-Gut-Mix as reference reagents for identifying biases in metagenomic analyses of multi-microbial communities. These reagents expose limitations across the entire analytical workflow, including sequencing depth, library preparation choices, and bioinformatic pipelines. Their application allows researchers to refine methodologies and minimize bias in microbiome profiling, particularly for both shotgun and 16S rRNA gene amplicon sequencing approaches. Additionally, we provide a valuable data set generated with these reagents that can serve as a resource for further meta-analyses and bioinformatic pipeline development.

The establishment of these reference reagents through the WHO, along with their rigorous evaluation and peer-reviewed documentation, ensures their credibility and accessibility for global use, while their relatively low cost facilitates widespread adoption. By incorporating these reagents into research protocols and publication guidelines, we can advance the standardization of microbiome research. This will ultimately enhance our understanding of the microbiome’s structure, evolution, and impact on human health. While future advancements may lead to even more complex quantitative standards, the WHO DNA-Gut-HiLo and WHO DNA-Gut-Mix RRs currently empower researchers to identify and address critical biases in microbiome profiling.

## MATERIALS AND METHODS

### Production of DNA reference reagents

The development of the reference reagents used in this study has been described in detail in reference 4. Briefly, strains (Table S1) were cultured individually as recommended by the supplier, the Leibniz Institute DSMZ (the German Collection of Microorganisms and Cell Cultures GmbH). DNA was extracted from each strain using the QIAGEN DNeasy Powersoil Kit (QIAGEN 12888). The extracted DNA from each strain was assessed for purity by Sanger and shotgun sequencing, as previously described by (4). Qubit fluorometer and TapeStation-automated electrophoresis were also employed to assess DNA quantity and integrity. The average DNA concentration for each strain was calculated using five independent DNA quantifications via Qubit double-stranded DNA High Sensitivity assay kit (Qubit dsDNA HS, Invitrogen). DNA from the 20 bacterial strains was pooled together at even concentrations for the DNA-Gut-Mix. For the DNA-Gut-HiLo reagent, the strain DNA concentration differed by three orders of magnitude, aiming to simulate the natural variability in microbial abundance (Table S5). The DNA mix was diluted to a concentration of 50 ng/µL in pure sterile water containing 2 mM Tris-HCl and 0.1% trehalose for the DNA-Gut-Mix (NIBSC 20/302) and in pure sterile water containing 2 mM Tris-HCl and 1% trehalose for the DNA-Gut-HiLo (NIBSC 20/304) with a final pH of 8.5. The material was filled at MHRA into 2.5 mL ampoules at 2°C–8°C with slow stirring, and vials were sealed and freeze-dried in a two-day cycle.

### Post-fill testing of DNA reference reagents

During the filling process, samples were retrieved from the beginning, middle, and end of the fill to ensure consistency among ampoules. Post-filling, DNA was quantified for five replicates of each test reagent by Qubit, and composition was assessed using shotgun sequencing (Illumina DNA prep kit, NextSeq 500, paired-end, 150 bp read length, and 10 million reads), followed by analysis using BBDuk (v.38.90) and MetaPhlAn3 (v3.0.9) (default settings). Sterility of reagents was tested using standard microbiological testing at MHRA. Owing to the very low dry weight, the residual moisture for NIBSC 20/302 could not be measured. The stability of the material was assessed by a long-term accelerated thermal degradation (ATD) study.

### Accelerated thermal degradation study

To assess the stability of the reference reagents across a range of temperatures over time, an ATD study was performed. Reagents were placed at five different temperatures: −70°C, −20°C, 4°C, 20°C, and 37°C. To date, samples from five time points have been assessed; 0.5, 1, 3, 6, and 12 months. For each time point, samples were blinded and randomized to avoid bias. At each time point, five replicates of each reagent were reconstituted with pure, sterile water, and metagenomic analysis of the microbiome composition was performed. The composition of the samples from each time point was analyzed using shotgun sequencing (employing the same approach as post-fill testing from 0.5 to 3 months, then changing only the sequencing platform to NextSeq2000). Stability was assessed by comparing reagent composition (similarity) to the baseline temperature of −20°C. Both RRs were found to be stable across all temperatures and time points up to 2 years (at time of writing), measuring above 98% similarity to baseline. Additional time points are set for 3, 5, and 10 years.

### Collaborative study design

A large international collaborative study was conducted to evaluate the suitability of the reagents as RRs for analysis of gut microbiome samples using NGS and to establish MQC for users. Participants were invited to independently test the RRs using their own in-house methods and/or an optional recommended method described by the MHRA, to perform shotgun sequencing and/or 16S rRNA gene amplicon sequencing. It is important to highlight that the MHRA-recommended method is not considered the best-performing method. An important factor of this study was to cover a range of methods used in the field across the world and across sectors; hence, the methods employed by the participants were not a selective criteria in the study design.

To ensure consistency in bioinformatics analysis when using the MHRA recommended method, full scripts were provided to participants (shotgun sequencing analysis: https://github.com/nibscbioinformatics/metagenomics_mpa3 and 16S rRNA gene amplicon sequencing analysis: https://github.com/nibscbioinformatics/ampliconseq_qiime2). Participants were asked to perform the sequencing and bioinformatics analysis for both DNA-Gut-Mix and DNA-Gut-HiLo RRs. Participants produced 19 shotgun sequencing data sets using 6 different sequencing platforms, 5 read lengths, 6 adapters, 14 library preparation protocols, and average read depth from 0.831 to 286 million reads per sample, while employing 6 different trimming tools, 9 distinct bioinformatic pipelines, and 9 databases (including different pipeline/tool combinations and versions), with and without rarefaction for their analyses. For the 23 16S rRNA gene amplicon sequencing data sets, participants used 4 different sequencing platforms, paired- and single-end reads, 5 read lengths, 8 different primer pairs, 15 DNA polymerases, 4 PCR cycle programs, 7 adapters, and 15 library preparation protocols with sequencing depths of 0.012 to 2.7 million reads per sample. These data sets were analyzed using 12 different trimming tools, 11 distinct pipelines, and 4 databases (again including different pipeline/tool combinations and versions), with and without rarefaction. It was suggested to analyze the data at the species level for shotgun sequencing and at the genus level for the 16S amplicon sequencing. The identity of the participants was not disclosed until the completion of the study to avoid any communication between the participants and hence any potential bias.

Five blinded ampoules of each reagent were shipped to participants: Sample A, the DNA-Gut-HiLo (20/304), and Sample B, the DNA-Gut-Mix (20/302). Participants were instructed to reconstitute the reagents with pure, sterile water, mix well, and process the samples on the same day. Following the conclusion of the study, blinded sequencing data were made available to all participants. A detailed summary of the methods used by participants can be found in Tables S1 and S7. The study was conducted in 2022, reflected in the versions of pipelines and tools used.

### Reporting system: four key reporting measures

Using the data generated from shotgun sequencing and 16S rRNA gene amplicon sequencing, measures of sensitivity, FPRA, diversity, and similarity based on the average abundances of the five replicates for each sample were calculated as previously described by (4). Sensitivity describes the percentage of correctly identified species or genera present in the RR. This measure is specific to metagenomic data and differs from the clinical definition of sensitivity, which measures the probability of identifying a true positive. FPRA reports the relative abundance (%) of species or genera identified that are false positives (i.e., percentage of identified species which were identified yet were not present in the reagent). Diversity describes the total number of all observed species (true positives and false positives). Reads not assigned a species or genus were excluded from the data to calculate the key reporting measures. Together, FPRA and diversity reflect the degree to which a given approach is too conservative (i.e., not identifying microbes that are present in the RRs), or less conservative (i.e., identifying false positives). Finally, similarity measures the similarity between the reported composition and the actual composition. We have created an R package for calculating the four key reporting measures, which can be found at https://github.com/nibscbioinformatics/microbiomeMQC.


$$\begin{array}{c} \text{ Sensitivity }=\frac{\text{ number of correctly identified microbes }}{\text{ total number of microbes in reagent }}\times 100,\\ \text{ false positive relative abundance }=\frac{\text{ abundance of false positive microbes }}{\text{ total abundance of all microbes }}\times 100,\\ \text{ diversity }=\text{ total number of all observed microbes }(\text{true positives }+\text{ false positives}),\\ \text{ similarity }[jk]=1-\frac{\mathrm{sumabs}(x[ij]-x[ik])}{\mathrm{sum}(x[ij]+x[ik])}. \end{array}$$

For sensitivity, FPRA, and diversity, the term “microbes” refers to either species or genera. For similarity, where x[ij] and x[ik] refer to the quantity of species/genera [i] in the actual composition [j] and observed composition [k] of the reagent. The similarity measure was calculated using 1 – Bray-Curtis dissimilarity. Bray-Curtis dissimilarity was calculated using the vegdist function of the vegan R package (method = “bray”).

### Calculation of the minimum quality criteria

The results of the four key reporting measures were shared with the participants and were used to calculate the MQC (Tables 1 and 4). As distributional assumptions could not be made regarding the four key reporting measures, a non-parametric statistical analysis was performed. For this analysis, we calculated the lower quartiles (LQ), upper quartiles (UQ) (i.e., 25% of the results are below LQ and 25% of results are above UQ), and the inter-quartile range (i.e., UQ–LQ, wherein 50% of all results lie), as these are typically used to summarize the distribution in non-parametric analyses.

To ensure an acceptable number of participants were able to achieve all four key reporting measures, the LQs were chosen as the MQC for the measures of sensitivity, diversity, and similarity, and the UQs for the FPRA, taking into consideration the optimal scores for each of the measures (Tables S2 and S8). The MQC was recalculated following the re-analysis of participant data published in the WHO ECBS report (9). The MQC values used in this work are also stated in the Instructions For Use for each reagent and should be used for the assessment of accuracy.

### Meta-analysis of the sequencing data

To separate sequencing and bioinformatics bias, participants were given access to all sequencing data sets produced during this study. Five participants conducted a meta-analysis of the shotgun sequencing data sets (Table 2), and eight did so for the 16S rRNA gene amplicon sequencing data sets (Table 4). Participants analyzed the sequencing data sets using different bioinformatic pipelines. Once again, this study was conducted in 2022, reflected in the versions of pipelines and tools used.

## Data Availability

All sequencing data generated throughout this study are publicly available on the NCBI Sequence Read Archive under the BioProject (PRJNA1238537). The R script for calculating the four key reporting measures can be found at https://github.com/nibscbioinformatics/microbiomeMQC. All abundance data generated through this study can be found at https://github.com/nibscbioinformatics/DNA-Reference-Reagents-Isolate-Biases-in-Microbiome-Profiling-A-Global-Multi-Lab-Study-Data. All reference reagents are available for purchase (https://www.nibsc.org/products.aspx).

## SUPPLEMENTAL MATERIAL

Supplemental material msystems.00466-25-s0001.pdf Supplemental figures and tables.

ASM does not own the copyrights to Supplemental Material that may be linked to, or accessed through, an article. The authors have granted ASM a non-exclusive, world-wide license to publish the Supplemental Material files. Please contact the corresponding author directly for reuse.
