# Supplementary material for: DNA reference reagents isolate biases in microbiome profiling: a global multi-lab study
Source: mSystems. 2025 Oct 17;10(11):e00466-25. doi: 10.1128/msystems.00466-25 (PMC12625696; doi:10.1128/msystems.00466-25)
Supplement: Supplemental material — Supplemental figures and tables. [file msystems.00466-25-s0001.pdf]

Supplementary Table 1 Strains used in NIBSC 20/302, DNA-Gut-Mix and NIBSC 20/304, DNA-Gut-HiLo and information on the composition of the reagents.

Strain number	Strain name	Genome size (bp)	16S rRNA gene copy number	DNA-Gut-Mix (20/302)			DNA-Gut-HiLo (20/304)		
				DNA added in RR (ng)	Genome size adjusted composition (%)	16S copy number adjusted composition (%)	DNA added in RR (ng)	Genome size adjusted composition (%)	16S copy number adjusted composition (%)
DSM 22959	*Akkermansia muciniphila*	2,664,102	3	5	6.4	4.65	0.625	0.18	0.12
DSM 17242	*Alistipes finegoldii*	3,734,239	2	5	4.5	2.21	6.25	1.3	0.55
DSM 3319	*Anaerostipes hadrus*	2,776,939	1	5	6.1	1.49	6.25	1.75	0.37
DSM 2079	*Bacteroides thetaiotaomicron*	6,293,399	5	5	2.7	3.28	62.5	7.72	8.22
DSM 6597	*Bacteroides uniformis*	4,634,992	4	5	3.7	3.56	6.25	1.05	0.89
DSM 20088	*Bifidobacterium longum subsp. infantis*	2,832,748	4	5	6.0	5.83	62.5	17.2	14.61
DSM 20219	*Bifidobacterium longum subsp. longum*	2,449,019	4	5	6.9	6.74	62.5	19.82	16.9
DSM 19850	*Blautia wexlerae*	4,510,428	2	5	3.8	1.83	0.625	0.11	0.05
DSM 10702	*Clostridium butyricum*	4,596,811	1	5	3.7	0.9	62.5	10.59	2.25
DSM 13712	*Collinsella aerofaciens*	2,439,869	7	5	7.0	11.85	6.25	1.99	2.97
DSM 1103	*Escherichia coli*	5,203,440	7	5	3.3	5.55	62.5	9.33	13.92
DSM 3353	*Eubacterium/Anaerobutyricum hallii*	3,290,996	1	5	5.2	1.25	6.25	1.48	0.31
DSM 17677	*Faecalibacterium praunitzii*	3,080,849	3	5	5.5	4.02	0.625	0.16	0.1
DSM 20077	*Lactobacillus gasseri/paragasseri*	1,894,360	6	5	9.0	13.08	0.625	0.26	0.33
DSM 20701	*Parabacteroides distasonis*	4,811,379	7	5	3.5	6.01	62.5	10.1	15.05
DSM 18205	*Prevotella copri*	3,507,873	7	5	4.8	8.24	62.5	13.84	20.65
DSM 7089	*Prevotella melaninogenica*	3,168,282	4	5	5.3	5.21	6.25	1.53	1.31
DSM 16839	*Roseburia hominis*	3,592,125	4	5	4.7	4.6	6.25	1.35	1.15
DSM 14610	*Roseburia intestinalis*	4,380,675	6	5	3.9	5.66	0.625	0.11	0.14
DSM 19829	*Ruminococcus gauvreauii*	4,096,474	4	5	4.1	4.03	0.625	0.12	0.1

Supplementary Table 2 Participant shotgun sequencing and bioinformatics methods.

Participant	Sequencing						Bioinformatics				
	Platform	Paired end	Read length (bp)	Adapters	Sequencing depth (reads per sample)	Library prep and sequencing protocol	Rcmd method used	Trimming	Pipeline	Database	Subsampling / rarefaction
Lab 2	MiSeq	Yes	150	Nextera Flex	2,631,337	Illumina DNA Prep Reference Guide (1000000025416 v09, June 2020)	Yes	BBDuk (v.38.90)	MetaPhlAn3 (v.3.0.9)	ChocoPhlAn3	Seqtk 1.3: 250k fwd, 250k rev
Lab 4	NovaSeq 6000	Yes	150	NEBNext® Ultra™ II FS DNA Library Prep Kit	43,886,507	NEBNext® Ultra™ II FS DNA Library Prep Kit	No	N/A	NGLess (v1.0.1)	mOTUs2 (v2.5.1)	No
Lab 5	MiSeq	Yes	250	NEBNext® Ultra™ II FS DNA Library Prep Kit	2,985,198	NEBNext® Ultra™ II FS DNA Library Prep Kit for Illumina® MiSeq v2 500 flow	No	CutAdapt	Kraken2 (v2.1.2)	centrifuge / kraken2	No
Lab 5	MiSeq	Yes	250	NEBNext® Ultra™ II FS DNA Library Prep Kit	2,985,198	NEBNext® Ultra™ II FS DNA Library Prep Kit for Illumina® MiSeq v2 500 flow	Yes	BBDuk (v.38.90)	MetaPhlAn3 (v.3.0.9)	ChocoPhlAn3	No
Lab 6	MiSeq	Yes	300	Nextera XT	8,194,529	Nextera Library Prep kit	No	CutAdapt	MetaHood	RefSeq and GTDB	No
Lab 8	NextSeq 500	Yes	150	Nextera Flex	1,720,647	DNA Prep protocol (Illumina® DNA Prep, (M) Tagmentation, Illumina, 20018704)	No	BBDuk (v.38.43)	MetaPhlAn3 (v.3.0.13)	ChocoPhlAn3	No
Lab 10	iSeq100	Yes	150	Nextera Flex	889,461	Nextera DNA prep (Illumina 20018705) V2 chemistry (Illumina 20031371)	No	CutAdapt	Kraken2 (v2.1.2)	NCBI (nt)	No
Lab 11	NovaSeq 6000	Yes	150	Nextera XT	79,810,099	Nextera Library Prep kit	No	CutAdapt	BURST (v0.99.7) / QIIME (v.1.9.1) / Prokka (v 1.12)	RefSeq / Venti (v0.1.3)	No
Lab 13	NextSeq 500	Yes	150	Nextera Flex	29,483,248	Illumina DNA Prep Reference Guide using the High Output kit	Yes	BBDuk (v.38.90)	MetaPhlAn3 (v.3.0.9)	ChocoPhlAn3	Seqtk 1.3: 250k fwd, 250k rev
Lab 14	NovaSeq 6000	Yes	150	Nextera XT	42,192,227	Illumina DNA Prep protocol	Yes	BBDuk (v.38.90)	MetaPhlAn3 (v.3.0.9)	ChocoPhlAn3	Seqtk 1.3: 250k fwd, 250k rev
Lab 15 - MiSeq	MiSeq	Yes	151	Nextera Flex	5,255,739	Illumina DNA Prep, #20018705	Yes	BBDuk (v.38.90)	MetaPhlAn3 (v.3.0.9)	ChocoPhlAn3	Seqtk 1.3: 250k fwd, 250k rev
Lab 15 - NS2000	NextSeq 2000	Yes	149	Nextera Flex	286,130,954	Illumina DNA Prep, #20018705	Yes	BBDuk (v.38.90)	MetaPhlAn3 (v.3.0.9)	ChocoPhlAn3	Seqtk 1.3: 250k fwd, 250k rev
Lab 15 - NS550	NextSeq 550	Yes	151	Nextera Flex	38,842,577	Illumina DNA Prep, #20018705	Yes	BBDuk (v.38.90)	MetaPhlAn3 (v.3.0.9)	ChocoPhlAn3	Seqtk 1.3: 250k fwd, 250k rev
Lab 16	MiSeq	Yes	150	NEBNext® Multiplex oligos for Illumina (Dual Index Primers Set 1)	3,850,221	NEBNext® Ultra™ II DNA library Prep Kit	No	Shi7 default parameters	SHOGUN pipeline (https://github.com/ knights-lab/SHOGUN)	Alignment: Bowtie2, Taxonomy: Default SHOGUN pipeline	No
Lab 18	NextSeq 550	Yes	150	Nextera XT	13,567,684	Illumina DNA Prep kit cat. no: 20018704 NextSeq 500/550 Mid Output Kit (v2.5) (300 Cycles) cat. no 20024905)	No	BBDuk (v.38.90)	KneadData (v0.7.4) / HUMAnN3 / MetaPhlAn3 (v3.0.7)	mpa v30 ChocoPhlAn (201901)	No
Lab 19	NovaSeq 6000	Yes	150	Nextera Flex	50,499,977	Illumina DNA Prep Reference Guide (Document # 1000000025416 v09, June 2020) Novaseq (v1.5) chemistry	No	Bcl2fastq Trimmomatic (v0.36) / BMTagger	MetaPhlAn3 (v.3.0.13)	ChocoPhlAn3	No
Lab 21	NextSeq 550	Yes	150	Nextera XT	831,031	Nextera DNA Prep Library Prep Kit (Illumina) (Cat no: 20018705)	No	FASTQC (v0.11.9)	DRAGEN (v.3.5.10)	Kraken2 (March 2020) database	No
Lab 22	MiSeq	Yes	300	NEBNext® Multiplex Oligos for Illumina® (Index Primers Sets 1 and 2)	4,063,198	NEBNext Ultra II FS DNA Library Prep Kit	No	Trimmomatic (v0.38)	MetaPhlAn3 (V.3.0.0) / bowtie2 / QIIME2 (v.2021.2)	mpa v30 ChocoPhlAn (201901)	No
Lab 24	NovaSeq 6000	Yes	150	Novogene adapters (Illumina compatible)	43,414,322	Novogene NGS DNA Library Prep Set. NovaSeq 6000 S4 Reagent Kit (v1.5)	No	N/A	MetaPhlAn3 (v.3.0.13)	ChocoPhlAn3	No

Supplementary Table 3 Participant 16S rRNA amplicon sequencing and bioinformatics methods. P-, paired-end and S-, single-end.

Participant	Sequencing									Bioinformatics				
	Platform	Paired end	Read length (bp)	Primer pair	DNA polymerase	PCR cycle No.	Adapters	Sequencing depth (avg reads per sample)	Library prep and sequencing protocol	Rcmd method used	Trimming	Pipeline	Database	Subsampling / rarefaction
Lab 2	MiSeq	Yes	300	V4: 515F/806R	Kapa HiFi Hot Start Ready Mix	25	Nextera Flex	301,750	Illumina 16S Metagenomic Sequencing Library guide (v. 15044223-B)	No	CutAdapt / DADA2 (v.1.20.0)	DADA2 (v.1.20.0) / R (v.4.1.0)	SILVA (v.138)	No
Lab 3	MiSeq	Yes	300	V3-V4: 319F/806R	FastStart High Fidelity Enzyme Blend	30	Integrated in PCR primers	194,050	Illumina 16S Metagenomic Sequencing Library guide	No	TagCleaner (v0.16)	R (v4.1.0) / DADA2	SILVA (v.132)	No
Lab 4	NovaSeq 6000	Yes	150	V4: undisclosed	Q5 High-Fidelity DNA Polymerase	8	Illumina barcoded adapters (NEBNext® Ultra™ II FS DNA Library Prep Kit)	2,704,435	NEBNext® Ultra™ II FS DNA Library Prep Kit	No	QIIME2 (v2020-8)	QIIME2 (v2020-8) / Deblur	SILVA (v.138)	No
Lab 5	MiSeq	Yes	250	V4: 515F/806R custom 44 base lead region	ThermoFisher Plat II Hot Start MMx2	35	Nextera XT	2,086,027	Earthmicrobiome protocol	No	CutAdapt (v2.1)	Nextflow (v.21.04.3) / nf-core ampliseq (v.2.0.0)	SILVA (v.138)	No
Lab 6	MinION	Paired and single	Long read	V1-V9: 27F/1492R	Q5 High-Fidelity DNA Polymerase (M0491)	25	AMII nanopore sequencing kit LSK 109	P- 472,216<br>S- 567,195	AMIIAMII	No	Guppy5	Minimap2 / Lowest Common Anecstor (LCA) alogrithm	SILVA (v.138)	No
Lab 7	MiSeq	Yes	250	V4: 515F/806R	Platinum Taq DNA Polymerase High Fidelity	30	Nextera XT	534,211	Illumina MiSeq v3 kit, 2x300bp kit	No	CutAdapt (v 3.4)	DADA2 (v.1.20.0) / R (v.4.1.0)	DECIPHER (v. 2.20.0) / SILVA (v.138)	No
Lab 8 - H	MiSeq	Yes	300	V4: 515F/806R	Kap2G Robust DNA Polymerase	30	Nextera XT	528,347	V3 600 cycle kit	No	CutAdapt (v 3.4)	Dadaist2 (v.1.1.0)	SILVA (v.138)	No
Lab 8 - R	MiSeq	Yes	300	V4: 515F/806R	Kap2G Robust DNA Polymerase	30	Nextera XT	528,347	V3 600 cycle kit	Yes	CutAdapt / QIIME2 (v.2020.2)	QIIME2 (v.2020.2) / Deblur	SILVA (v.138)	No
Lab 9	MiSeq	Yes	300	V3-V4: 341F/805R	Kapa HiFi Hot Start Ready Mix	25	Nextera XT	308,367	Illumina 16S Metagenomic Sequencing Library guide (v. 15044223-B)	No	Trimmomatic (v.0.39)	DADA2 / R (v.4.1.1)	SILVA (v.138.1)	No
Lab 11	MiSeq	Yes	250	V4: 515F/806R	5 PRIME HotMasterMix	35	Nextera XT	617,635	Earthmicrobiome protocol using MiSeq Reagent Kit V3 (600 cycle)	No	DADA2 (v1.16.0) / QIIME (1.9.1)	DADA2 (v.1.16.0) / QIIME (1.9.1)	SILVA (v.138)	No
Lab 12	MiSeq	Yes	250	V3-V4: 341F/805R	Super FI Platinum enzyme	35	Nextera Flex	85,285	Illumina 16S Metagenomic Sequencing Library guide (v. 15044223-B)	No	QIIME 2 (v.2020.6) / DADA2	QIIME2 (v.2020.6) / DADA2	SILVA (v.132)	2000 reads per sample
Lab 13	MiSeq	Yes	250	V4: 515F/806R	ThermoFisher Platinum Hot Start	35	Nextera Flex	316,174	Illumina 16S Metagenomic Sequencing Library guide (v. 15044223-B)	Yes	CutAdapt / QIIME2 (v.2020.2)	QIIME2 (v.2020.2) / Deblur	SILVA (v.138)	No
Lab 14	MiSeq	Yes	300	V3-V4: 341F/806R	Kapa HiFi Hot Start Ready Mix	25	Nextera XT	220,005	Illumina v3 2x300 bp sequencing kit	Yes	CutAdapt / QIIME2 (v.2020.2)	QIIME2 (v.2020.2) / Deblur	SILVA (v.138)	No
Lab 16	MiSeq	Yes	300	V1-V2: 28F-YM:28F-388R Borrellia:28F-388R Chloroflex: 28F-388RBifdo at a ratio of 4:1:1:1	Kapa ready mix hotstart hi-fi polymerase	30	Nextera XT	146,041	Illumina's 16S Metagenomic Sequencing Library Preparation Protocol and a MiSeq 2 x 300-cycle v3 kit	No	DADA2 (v1.8)	DADA2 (v.1.8) / R	SILVA (v.138.1)	No
Lab 17	MiSeq	Yes	250	V4: 515F/806R	Accuprime HiFi polymerase	25	Integrated in PCR primers	571,525	Illumina 16S Metagenomic Sequencing Library guide and a 2x 300-cycle MiSeq® Reagent Kit v3	No	Biostrings (v.2.60.0)	DADA2 / R (v.4.1.0)	SILVA (v.138.1)	No
Lab 18	MiSeq	Yes	250	V4: 515F/806R	Phusion Hot Start II High Fidelity DNA Polymerase	30	Nextera XT	1,209,969	V3 MiSeq reagent kit (600 cycles)	No	DADA2 (v.1.16.0)	DADA2 (v.1.16.0)	SILVA (v.138.1)	No
Lab 20	iSeq 100	Yes	301	V4: 515F/806R	KAPA HiFi HotStart ReadyMix	25	Nextera XT	317,345	Nextera XT DNA Library Preparation Kit with the sequencing cartridge (iSeq 100 Reagents v2)	No	Trimmomatic (v0.32) / EzBioCloud (210707)	HMMER's hmmsearch (v.3.3.2) / USEARCH (8.1.1861_i86linux32) / EzBioCoud (210707)	EzBioCloud 16S (v.PKSSU4.0)	No
Lab 21	iSeq 100	Yes	150	V3-V4: 341F/805R	KAPA HiFi HotStart ReadyMix	25	Nextera XT	168,015	Illumina's 16S Metagenomic Sequencing Library guide (v. 15044223)	No	Trimmomatic (v.032)	Ribosomal Database Project (RDP) Classifier (v.11.1)	Greengenes	No
Lab 22	MiSeq	Yes	300	V4: 515F/806R	FastStart High Fidelity Enzyme Blend	30	NEBNext Adaptor for Illumina (primer set 1 and 2)	189,625	v3 (600) flow cell for MiSeq desktop sequencer (Illumina)	No	QIIME2 (v.2021.2) / DADA2	QIIME2 (v.2021.2) / DADA2	SILVA (v.138)	No
Lab 23	MiSeq	Yes	300	V4: 515F/806R	DreamTaq Green PCR Master Mix	30	Earth Microbiome Project adapters	11,755	TrueSeq Nano DNA Library Prep Kit.	No	DADA2 / R (v.1.18.0)	DADA2 / R (v.1.18.0)	SILVA (v.138.1)	No
Lab 24 - H - V4	MiSeq	Yes	300	V4: 515F/806R	Phusion High-Fidelity PCR Master Mix	25	Nextera XT	559,605	Illumina MiSeq® Reagent Kit v3	No	CutAdapt / DADA2	DADA2 custom	SILVA (v.138)	No
Lab 24 - H - V3-V4	MiSeq	Yes	300	V3-V4: 341F/805R	Phusion High-Fidelity PCR Master Mix	25	Nextera XT	354,311	Illumina MiSeq® Reagent Kit v3	No	CutAdapt / DADA2	DADA2 custom	SILVA (v.138)	No

Supplementary Table 4 Participants to reach the MQC for all key reporting measures for both DNA-Gut-HiLo and DNA-Gut-Mix reference reagents following shotgun sequencing data analysis. Participants in bold achieved the ground truth for the given reporting measure.

Key reporting measures	RR	No. of participants	Participant above MQC
All key reporting measures	HiLo	9	2, 8, 10, 13, 15-NS2000, 18, 19, 22, 24
	Mix	10	8, 10, 13, 14, 15-MiSeq, 15-NS2000, 15-NS550, 18, 22, 24
	Both	7	8, 10, 13, 15-NS2000, 18, 22, 24

Key reporting measures	RR	No. of participants	Participant above MQC	No. of participants	Participant below MQC
Sensitivity	HiLo	15	2, 4, 5-H, 6, 8, 10, **11**, 13, 15-NS2000, 16, 18, 19, 21, 22, 24	4	5-R, 14, 15-NS550, 15-MiSeq
	Mix	15	4, 6, 8, 10, **11**, 13, 14, 15-NS550, 15-NS2000, 15-MiSeq, 16, 18, 19, 22, 24	4	2, 5-H, 5-R, 21
FPRA	HiLo	14	**2, 5-H, 5-R, 8, 10, 13, 14, 15-NS550, 15-NS2000, 15-MiSeq, 18, 19, 22, 24**	5	4, 6, 11, 16, 21
	Mix	14	**2, 5-H, 5-R, 8, 10, 13, 14, 15NS550, 15NS2000, 15MiSeq, 18, 19, 22, 24**	5	4, 6, 11, 16, 21
Diversity	HiLo	11	2, 5-H, **6**, 8, 10, 13, 15-NS2000, **18**, **19**, 22, **24**	8	4, 5-R, 11, 14, 15-NS550, 15-MiSeq, 16, 21
	Mix	11	**6**, 8, 10, 13, 14, 15-NS550, 15-NS2000, 15-MiSeq, **18**, **22**, **24**	8	2, 4, 5-H, 5-R, 11, 16, 19, 21
Similarity	HiLo	14	2, 4, 5-R, 8, 10, 13, 14, 15-NS550, 15-NS2000, 15-MiSeq, 18, 19, 22, 24	5	5-H, 6, 11, 16, 21
	Mix	14	2, 4, 5-R, 8, 10, 13, 14, 15NS550, 15-NS2000, 15-MiSeq, 18, 19, 22, 24	5	5-H, 6, 11, 16, 21

Supplementary Table 5 Participants to reach the MQC for each and all of the key reporting measures for both the DNA-Gut-HiLo and DNA-Gut-Mix reference reagents following 16S rRNA amplicon sequencing data analysis. Participants in bold achieved the ground truth for the given reporting measure.

Key reporting measures	RR	No. of participants	Participant
All key reporting measures	HiLo	5	5, 8-R, 13, 14, 23
	Mix	4	8-R, 13, 14, 23
	Both	4	8-R, 13, 14, 23

Key reporting measures	RR	No. of participants	Participant above MQC	No. of participants	Participant below MQC
Sensitivity	HiLo	18	**2**, 4, 5, **6-P**, **6-S**, **7**, 8-R, **9**, 11, 13, 14, **16**, **17**, 18, **20**, **21**, 22, 23	5	3, 8-H, 12, 24-V3-V4, 24-V4
	Mix	18	**2**, 4, 5, **6-P**, **6-S**, **7**, 8-R, **9**, 11, 13, 14, **16**, **17**, 18, **20**, **21**, 22, 23	5	3, 8-H, 12, 24-V3-V4, 24-V4
FPRA	HiLo	17	2, 4, 5, 7, 8-R, 9, **11**, 12, 13, 14, 17, 18, 20, 22, 23, **24-V3-V4, 24-V4**	6	2, 3, 6-P, 6-S, 8-H, 21
	Mix	17	2, 4, 5, 6-P, 6-S, 8-R, **9**, **11**, 13, 14, 16, 17, **18**, 19, 22, 23, 24-V3-V4, **24-V4**	6	3, 5, 7, 8-H, 12, 21
Diversity	HiLo	6	**5**, **8-R**, **13**, **14**, **22**, **23**	17	2, 3, 4, 6-P, 6-S, 7, 8-H, 9, 11, 12, 16, 17, 18, 20, 21, 24-V3-V4, 24-V4
	Mix	6	**5**, **8-R**, **13**, **14**, **22**, **23**	17	2, 3, 4, 6-P, 6-S, 7, 8-H, 9, 11, 12, 16, 17, 18, 20, 21, 24-V3-V4, 24-V4
Similarity	HiLo	19	2, 4, 5, 7, 8-H, 8-R, 9, 11, 13, 14, 16, 17, 18, 20, 21, 22, 23, 24-V3-V4, 24-V4	4	3, 6-P, 6-S, 12
	Mix	18	2, 4, 5, 6-P, 6-S, 7, 8-H, 8-R, 9, 11, 13, 14, 16, 17, 18, 20, 21, 23, 24-V4	5	3, 7, 12, 22, 24-V3-V4
Similarity (16S gene copy number adjusted)	HiLo	17	2, 3, 4, 5, 7, 8-H, 8-R, 9, 11, 13, 14, 16, 17, 18, 20, 21, 23, 24-H, 24-V4	6	3, 6-P, 6-S, 12, 22, 24-V3-V4
	Mix	18	2, 4, 5, 6-P, 6-S, 8-H, 8-R, 9, 11, 13, 14, 16, 17, 18, 20, 21, 23, 24-V3-V4	5	3, 7, 12, 22, 24-V4

Supplementary Table 6 Coefficient of variation (CV) across all participants for each key reporting measure for both shotgun and 16S rRNA gene amplicon sequencing datasets and DNA-Gut-HiLo and DNA-Gut-Mix reference reagents (RR).

Key Reporting measure	Sensitivity		FPRA		Diversity		Similarity		Similarity (16S gene copy number adjusted)	
RR	HiLo	Mix	HiLo	Mix	HiLo	Mix	HiLo	Mix	HiLo	Mix
Shotgun CV (%)	17.2	5.9	215.6	318.9	142.1	125.4	13.2	15.5	-	-
16S CV (%)	5.8	4.7	145.9	102.5	106.2	102.3	15.8	8.9	17.3	9.7

Supplementary Table 7 Post-rarefaction key reporting measures of shotgun sequencing datasets. The ↓ suffix denotes a reduced measure. -R, recommended and -H, in-house.

Measure	Sensitivity (%)		FPRA (%)		Diversity		Similarity (%)	
Reference reagent	HiLo	Mix	HiLo	Mix	HiLo	Mix	HiLo	Mix
Actual	100	100	0	0	19	19	100	100
Lab2 - R	68	89	0	0	13	17	90	84
Lab4 - R	95	95	8	3	21	21 ↓	80	82
Lab5 - H	74	74	0	0	14	14	71	68
Lab5 - R	63	89	0	0	12	17	83	80
Lab6 - H	95	95	13	3	19	19	60	71
Lab8 - R	95	95	0	0	18	18	78	72
Lab10 - R	68 ↓	95	0	0	13 ↓	18	87	84
Lab11 - H	100	100	27	24 ↓	34 ↓	39 ↓	60	61
Lab13 - R	68	95	0	0	13	18	85	83
Lab14 - H	63	95	0	0	12	18	95	86
Lab15 - NS550	63	95	0	0	12	18	83	81
Lab15 - NS2000	68	95	0	0	13	18	89	85
Lab15 - MiSeq	63	89 ↓	0	0	12	17 ↓	88	84
Lab16 - H	95	95	7	41	80 ↓	74 ↓	55	34
Lab18 - R	95	95	0	0	18	18	88	85
Lab19 - R	95	95	0	0	18 ↓	18 ↓	91	86
Lab21 - H	84	84	2	4	60 ↓	101 ↓	68	66
Lab22 - H	89 ↓	95	37	36	17	18	63	63
Lab24	95	95	0	0	18 ↓	18 ↓	90	87

Supplementary Table 8 Key reporting measures of each participant shotgun sequencing dataset for both the DNA-Gut-HiLo and DNA-Gut-Mix RRs. Data are presented as distance from the MQC, with the 'optimal' values stated for each key reporting measure based on the distance of the ground truth from the MQC. Blue values met the MQC while orange/yellow values did not. -R, recommended and -H, in-house.

Participant	Sensitivity		FPRA		Diversity		Similarity		Participant shotgun sequencing and bioinformatics approaches							
	HiLo	Mix	HiLo	Mix	HiLo	Mix	HiLo	Mix	Sequecing platform	Read length (bp)	Adapters	Sequencing depth (per sample)	Trimming	Pipeline	Database	Sub-sampling (reads)
Optimal	32	5	-0.53	-1.29	0	0	25	28								
Lab2	0	-6	-0.53	-1.29	-6	-2	15	12	MiSeq	150	Nextera Flex	2,631,337	BBDuk	MetaPhlAn3	ChocoPhlAn3	500,000
Lab4	27	0	7.53	1.28	2	4	5	10	NovaSeq 6000	150	NEBNext Ultra II FS	43,886,507	N/A	NGLess	mOTUs2	No
Lab5 - H	6	-21	-0.53	-1.29	-5	-5	-4	-4	MiSeq	250	NEBNext Ultra II FS	2,985,198	CutAdapt	Kraken2	Centrifuge / kraken2	No
Lab5 - R	-5	-6	-0.53	-1.29	-7	-2	8	8	MiSeq	250	NEBNext Ultra II FS	2,985,198	BBDuk	MetaPhlAn3	ChocoPhlAn3	No
Lab6	27	0	12.83	1.96	0	0	-15	-1	MiSeq	300	Nextera XT	8,194,529	CutAdapt	MetaHood	RefSeq / GTDB	No
Lab8	27	0	-0.53	-1.29	-1	-1	3	0	NextSeq 500	150	Nextera Flex	1,720,647	BBDuk	MetaPhlAn3	ChocoPhlAn3	No
Lab10	6	0	-0.53	-1.29	-5	-1	12	12	iSeq100	150	Nextera Flex	889,461	CutAdapt	Kraken2	NCBI NT	No
Lab11	32	5	0.53	1.55	151	161	-6	-4	NovaSeq 6000	150	Nextera XT	79,810,099	CutAdapt	BURST / QIIME / Prokka	RefSeq / Venti	No
Lab13	0	0	-0.53	-1.29	-6	-1	10	11	NextSeq 500	150	Nextera Flex	29,483,248	BBDuk	MetaPhlAn3	ChocoPhlAn3	500,000
Lab14	-5	0	-0.53	-1.29	-7	-1	20	14	NovaSeq 6000	150	Nextera XT	42,192,227	BBDuk	MetaPhlAn3	ChocoPhlAn3	500,000
Lab15 - NS550	-5	0	-0.53	-1.29	-7	-1	8	13	NextSeq 550	149	Nextera Flex	38,842,577	BBDuk	MetaPhlAn3	ChocoPhlAn3	500,000
Lab15 - NS2000	0	0	-0.53	-1.29	-6	-1	14	9	NextSeq 2000	151	Nextera Flex	286,130,954	BBDuk	MetaPhlAn3	ChocoPhlAn3	500,000
Lab15 - MiSeq	-5	0	-0.53	-1.29	-7	-1	13	12	MiSeq	151	Nextera Flex	5,255,739	BBDuk	MetaPhlAn3	ChocoPhlAn3	500,000
Lab16	27	0	6.76	39.53	166	135	-20	-38	MiSeq	150	NEBNext Multiplex	3,850,221	Shi7	SHOGUN	RefSeq (Rep 82)	No
Lab18	27	0	-0.53	-1.29	0	0	13	13	NextSeq 550	150	Nextera XT	13,567,684	BBDuk	KneadData / HUMAnN3 / MetaPhlAn3	ChocoPhlAn3	No
Lab19	27	0	-0.53	-1.29	0	2	16	14	NovaSeq 6000	150	Nextera Flex	50,499,977	Bcl2fastq	MetaPhlAn3	ChocoPhlAn3	No
Lab21	16	-11	1.58	3.17	52	93	-3	-1	NextSeq 550	150	Nextera XT	831,031	Trimmom-atic	DRAGEN	Kraken2	No
Lab22	27	0	-0.53	-1.29	-1	0	2	5	MiSeq	300	NEBNext Multiplex (1&2)	4,063,198	FASTQC	MetaPhlAn3 / bowtie2 / QIIME2	ChocoPhlAn3	No
Lab24	27	0	-0.53	-1.29	0	0	15	15	NovaSeq 6000	150	Novogene adapters	43,414,322	Trimmom-atic	MetaPhlAn3	ChocoPhlAn3	No

Supplementary Table 9 Performance based on the MQC along with the metadata for 16S rRNA gene amplicon sequencing and bioinformatics approaches for both the DNA-Gut-HiLo and DNA-Gut-Mix RRs. Data are presented as distance from the MQC, with the 'optimal' values stated for each key reporting measure based on the distance of the ground truth from the MQC. Blue values met the MQC while orange/yellow values did not. -R, recommended, -H, in-house, -P, paired-end and -S, single-end.

Participant	Sensitivity		FPRA		Diversity		Similarity		Similarity (16S gene copy number adjusted)		Participant 16S rRNA amplicon sequencing and bioinformatics approaches							
	HiLo	Mix	HiLo	Mix	HiLo	Mix	HiLo	Mix	HiLo	Mix	Sequecing platform	Read length (bp)	Primers	Sequencing depth (per sample)	Trimming	Pipeline	Database	Sub-sampling (reads)
Optimal	6	6	-0.43	-4.08	0	0	45	32	43	39								
Lab 2	6	6	0.22	-3.58	2	2	6	10	11	11	MiSeq	300	V4: 515F/806R	301,750	CutAdapt / DADA2 (v.1.20.0)	DADA2 (v.1.20.0) / R (v.4.1.0)	SILVA (v.138)	No
Lab 3	-13	-6	1.48	7.00	1	8	-12	-8	-8	-8	MiSeq	300	V3-V4: 319F/806R	194,050	tagcleaner (v0.16)	R (v4.1.0) / DADA2	SILVA (v.132)	No
Lab 4	0	0	-0.30	-0.68	5	4	16	12	15	8	NovaSeq 6000	150	V4: undisclosed	2,704,435	QIIME 2 (v2020-8)	QIIME2 / Deblur	SILVA (v.138)	No
Lab 5	0	0	-0.31	0.02	0	0	9	5	9	0	MiSeq	250	V4: 515F/806R custom 44 bp lead region	2,086,027	CutAdapt (v2.1)	Nextflow (v.21.04.3) / nf-core ampliseq (v.2.0.0)	SILVA (v.138)	No
Lab 6 - P	6	6	0.21	-0.70	21	37	-17	0	-15	2	MinION	Long read	V1-V9: 27F/1492R	472,216	Guppy5	Minimap2	SILVA (v.138)	No
Lab 6 - S	6	6	0.23	-0.62	23	40	-17	0	-14	2	MinION	Long read	V1-V9: 27F/1492R	567,195	Guppy5	Minimap2	SILVA (v.138)	No
Lab 7	6	6	-0.38	5.34	10	29	0	-4	2	-6	MiSeq	250	V4: 515F/806R	534,211	CutAdapt (v 3.4)	DADA2 (v.1.20.0) / R (v.4.1.0)	DECIPHER (v. 2.20.0) / SILVA (v.138)	No
Lab 8 - H	-6	-6	0.86	2.31	1	-1	11	9	17	10	MiSeq	300	V4: 515F/806R	528,347	CutAdapt (v 3.4)	Dadaist2 (v.1.1.0)	SILVA (v.138)	No
Lab 8 - R	0	0	-0.33	-1.04	0	0	12	13	17	11	MiSeq	300	V4: 515F/806R	528,347	CutAdapt / QIIME2	QIIME2 (v.2020.2) / Deblur	SILVA (v.138)	No
Lab 9	6	6	-0.42	-4.07	7	6	5	12	10	12	MiSeq	300	V3-V4: 341F/805R	308,367	Trimmomatic (v.0.39)	DADA2 / R (v.4.1.1)	SILVA (v.138.1)	No
Lab 11	0	0	-0.43	-4.08	3	5	10	12	14	13	MiSeq	250	V4: 515F/806R	617,635	DADA2 (v1.16.0) / QIIME (1.9.1)	DADA2 (v1.16.0) / QIIME (1.9.1)	SILVA (v.138)	No
Lab 12	-6	-6	-0.22	6.43	2	7	-8	-6	-16	-4	MiSeq	250	V3-V4: 341F/805R	85,285	QIIME 2 (v.2020.6) / DADA2	QIIME 2 (v.2020.6) / DADA2	SILVA (v.132)	2000 (reads per sample)
Lab 13	0	0	-0.27	-0.75	0	0	13	11	8	6	MiSeq	250	V4: 515F/806R	316,174	CutAdapt / QIIME2	QIIME2 (v.2020.2) / Deblur	SILVA (v.138)	No
Lab 14	0	0	-0.31	-1.35	0	0	8	5	12	6	MiSeq	300	V3-V4: 341F/806R	220,005	CutAdapt / QIIME2	QIIME2 (v.2020.2) / Deblur	SILVA (v.138)	No
Lab 16	6	6	-0.41	-4.05	5	10	3	8	9	8	MiSeq	300	V1-V2: 28F-YM:28F-Borrellia:28F-Chloroflex: 28F-Bifdo 4:1:1:1	146,041	DADA2 (v1.8)	DADA2 (v.1.8) / R	SILVA (v.138.1)	No
Lab 17	6	6	-0.41	-4.07	14	9	11	12	15	10	MiSeq	250	V4: 515F/806R	571,525	Biostrings (v.2.60.0)	DADA2 / R (v.4.1.0)	SILVA (v.138.1)	No
Lab 18	0	0	-0.40	-4.08	6	1	1	9	11	6	MiSeq	250	V4: 515F/806R	1,209,969	DADA2 (v.1.16.0)	DADA2 (v.1.16.0)	SILVA (v.138.1)	No
Lab 20	6	6	-0.07	-0.01	12	12	3	6	5	4	iSeq 100	301	V4: 515F/806R	317,345	Trimmomatic (v0.32) / EzBioCloud (210707)	hmmsearch (v.3.3.2) / USEARCH (8.1.1861_i86linux32) / EzBioCoud (210707)	EzBioCloud 16S (v.PKSSU4.0)	No
Lab 21	6	6	1.05	0.06	141	145	8	9	13	11	iSeq 100	150	V3-V4: 341F/805R	168,015	Trimmomatic (v.032)	RDP Classifier (v.11.1)	Greengenes	No
Lab 22	0	0	-0.41	-2.49	0	0	0	-4	-9	-2	MiSeq	300	V4: 515F/806R	189,625	QIIME2 (v.2021.2) / DADA2	QIIME2 (v.2021.2) / DADA2	SILVA (v.138)	No
Lab 23	0	0	-0.26	-1.49	0	0	5	6	4	0	MiSeq	300	V4: 515F/806R	11,755	DADA2 in R (v.1.18.0)	DADA2 in R (v.1.18.0)	SILVA (v.138.1)	No
Lab 24 - V3-V4	-6	0	-0.43	-4.07	-1	1	0	-4	-9	-6	MiSeq	300	V3-V4: 341F/805R	354,311	CutAdapt / DADA2	DADA2 custom	SILVA (v.138)	No
Lab24 - V4	-6	-6	-0.43	-4.08	-2	-2	16	3	2	-1	MiSeq	300	V4: 515F/806R	559,605	CutAdapt / DADA2	DADA2 custom	SILVA (v.138)	No

Supplementary Table 10 A comparison of reported relative abundance (%) of genera in the DNA-Gut-HiLo and DNA-Gut-Mix RRs by Lab 16 using SILVA v.138 and SILVA v.138.1 databases following 16S rRNA amplicon sequencing. *Genera not present in the reference reagents.

Genera	DNA-Gut-HiLo			DNA-Gut-Mix		
	Actual	SILVA v.138	SILVA v.138.1	Actual	SILVA v.138	SILVA v.138.1
Akkermansia	0.18	0.031	0.031	6.37	1.744	1.744
Alistipes	1.3	0.322	0.322	4.54	1.556	1.557
Anaerostipes	1.75	1.400	1.400	6.11	5.205	5.205
Bacteroides	8.77	8.244	8.244	6.35	7.105	7.105
Bifidobacterium	37.02	14.118	14.118	12.92	7.305	7.304
Blautia	0.11	0.128	0.128	3.77	6.689	6.689
Clostridium	10.59	31.726	31.730	3.7	13.330	13.330
Collinsella	1.99	0.405	0.405	6.95	2.033	2.033
Escherichia	9.33	15.773	15.775	3.26	7.816	7.816
Eubacterium	1.48	0	1.308	5.16	0	5.317
Faecalibacterium	0.16	0.180	0.180	5.49	6.405	6.406
Lactobacillus	0.26	0.243	0.241	8.97	9.866	9.871
Parabacteroides	10.1	6.971	6.971	3.52	3.202	3.202
Prevotella	15.37	18.259	18.261	10.17	13.507	13.507
Roseburia	1.46	0.827	0.827	8.6	6.595	6.597
Ruminococcus	0.12	0	0.030	4.13	0	0.924
Actinomyces*	0	0	0	0	0.001	0.001
Cutibacterium*	0	0.004	0.004	0	0.013	0.013
Enterococcus*	0	0.003	0.002	0	0.003	0.003
Erysipelotrichaceae UCG-003*	0	0	0	0	0.001	0.001
Haemophilus*	0	0	0	0	0.001	0.001
Hafnia-Obesumbacterium*	0	0	0	0	0.001	0.001
Incertae Sedis*	0	0	0	0	0.001	0.001
Lachnospiraceae UCG-004*	0	0	0	0	0	0.001
Limosilactobacillus*	0	0	0.002	0	0	0
Peptoniphilus*	0	0	0	0	0.001	0.001
Pseudocitrobacter*	0	0	0	0	0.001	0
Pseudomonas*	0	0	0.001	0	0	0
Staphylococcus*	0	0.011	0.011	0	0.002	0.002
Unknown*	0	1.356	0.011	0	7.614	1.365

Supplementary Table 11 Lab 16 16S rRNA amplicon sequencing data key reporting measures comparison for both DNA-Gut-Hilo and DNA-Gut-Mix RRs when using SILVA v.138 and SILVA v.138.1.

Key reporting measure	Sensitivity (%)		FPRA (%)		Diversity		Similarity (%)		Similarity (16S-copy number adjusted) (%)	
**RR**	**HiLo**	**Mix**	**HiLo**	**Mix**	**HiLo**	**Mix**	**HiLo**	**Mix**	**HiLo**	**Mix**
**Actual**	100	100	0	0	16	16	100	100	100	100
**SILVA v.138**	87.5	87.5	1.37	7.64	18	25	68	69	66	66
**SILVA v.138.1**	100	100	0.03	1.39	22	27	69	75	66	69

Supplementary Table 12 Lab 13 comparison of meta-analyses of non-rarefied and rarefied 16S rRNA amplicon sequencing datasets by way of the four key reporting measures.

Rarefaction	Non-rarefied										Rarefied									
Key reporting measure	Sensitivity (%)		FPRA (%)		Diversity		Similarity (%)		Similarity (16S copy number adjusted) (%)		Sensitivity (%)		FPRA (%)		Diversity		Similarity (%)		Similarity (16S copy number adjusted) (%)	
RR	HiLo	Mix	HiLo	Mix	HiLo	Mix	HiLo	Mix	HiLo	Mix	HiLo	Mix	HiLo	Mix	HiLo	Mix	HiLo	Mix	HiLo	Mix
Lab 2	94	94	0	3	17	17	73	74	67	69	94	94	0	3	17	17	77	74	67	69
Lab 3	94	94	0	5	17	17	71	68	62	57	94	94	0	5	17	17	73	71	62	57
Lab 4	94	94	0	3	16	16	80	79	73	70	94	94	0	3	16	16	84	76	73	70
Lab 5	94	94	0	4	16	16	72	73	66	61	94	94	0	4	16	16	77	75	66	61
Lab 7	94	94	0	27	22	22	61	57	56	51	94	94	0	27	20	21	67	57	56	51
Lab 8	94	94	0	3	16	16	64	71	60	66	94	94	0	3	16	16	72	71	60	66
Lab 9	94	94	0	3	17	17	77	80	71	70	94	94	0	3	17	17	81	79	71	70
Lab 11	94	94	0	2	16	16	79	80	73	75	94	94	0	2	16	16	84	75	73	75
Lab 12	94	94	1	4	20	19	62	72	57	61	94	94	1	4	21	20	68	74	57	61
Lab 13	94	94	0	3	16	16	72	79	65	67	94	94	0	3	16	16	77	76	65	67
Lab 14	94	94	0	3	17	17	74	73	73	68	94	94	0	3	17	17	82	77	73	68
Lab 16	94	100	0	1	16	17	62	65	64	61	94	100	0	1	16	17	69	68	64	60
Lab 17	94	94	0	3	17	17	80	79	75	69	94	94	0	3	17	17	86	79	75	69
Lab 18	94	94	0	4	16	16	68	72	67	65	94	94	0	4	16	16	72	70	67	65
Lab 20	100	100	0	5	26	26	64	67	58	59	100	100	0	5	26	26	69	65	57	59
Lab 21	-	-	-	-	-	-	-	-	-	-	-	-	-	-	-	-	-	-	-	-
Lab 22	94	94	0	4	16	16	63	69	57	61	94	94	0	4	16	16	70	67	57	61
Lab 23	75	94	0	1	12	16	44	50	36	44	63	94	0	1	10	16	45	47	35	43
Lab 24-H-V3V4	88	94	1	3	21	19	54	68	50	57	88	94	1	3	22	20	60	67	50	57
Lab 24-H-V4	94	94	0	2	16	16	70	72	58	63	94	94	0	2	16	16	64	67	58	63
MQC	≥93.75	≥93.75	≤0.79	≤4.14	=16	=16	≥58	≥68	≥49	≥59	≥93.75	≥93.75	≤0.79	≤4.14	=16	=16	≥58	≥68	≥49	≥59

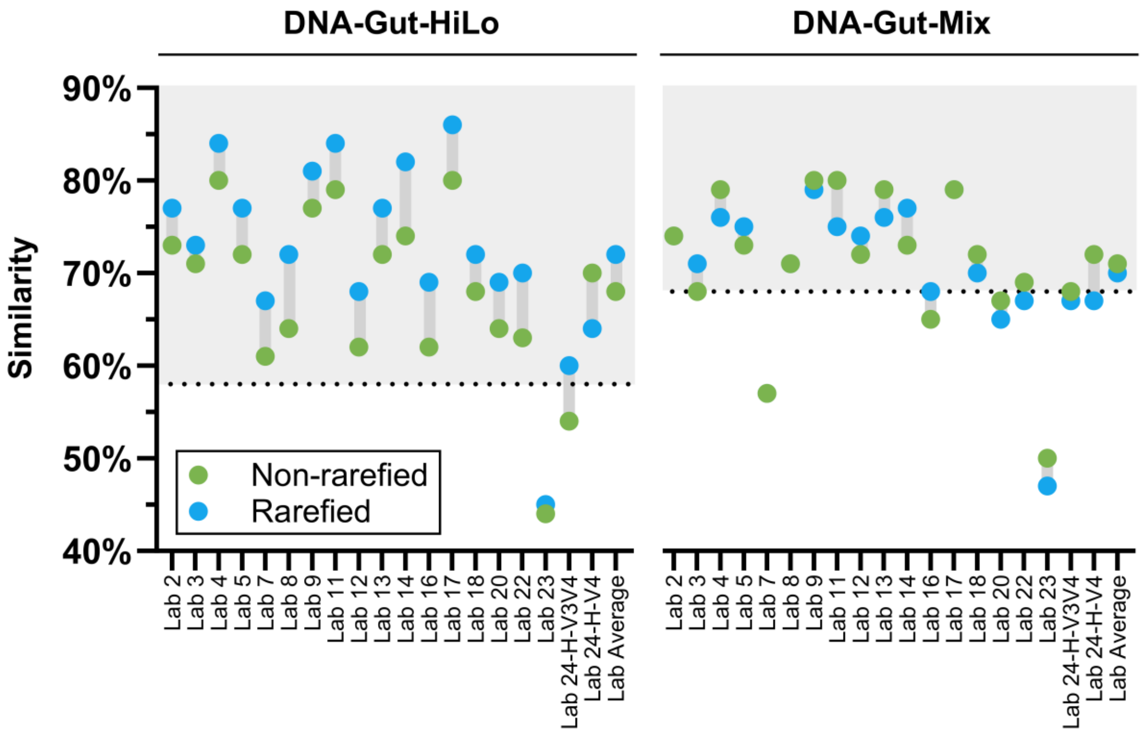

Supplementary Fig. 1 The effects of rarefication of 16S rRNA sequencing datasets on the measure of Similarity for both DNA-Gut-HiLo and DNA-Gut-Mix RRs through meta-analyses conducted by Lab 13. Data within the grey area are above the MQC. Where only non-rarefied data are visible both similarity measures are equal and hence overlap.
